# A Real-Time ITS1-PCR Based Method in the Diagnosis and Species Identification of *Leishmania* Parasite from Human and Dog Clinical Samples in Turkey

**DOI:** 10.1371/journal.pntd.0002205

**Published:** 2013-05-09

**Authors:** Seray Ozensoy Toz, Gulnaz Culha, Fadile Yıldız Zeyrek, Hatice Ertabaklar, M. Ziya Alkan, Aslı Tetik Vardarlı, Cumhur Gunduz, Yusuf Ozbel

**Affiliations:** 1 Ege University, Medical School Department of Parasitology, Bornova, Izmir, Turkey; 2 Mustafa Kemal University, Medical School Department of Parasitology, Hatay, Turkey; 3 Harran University, Medical School Department of Microbiology, Sanliurfa, Turkey; 4 Adnan Menderes University, Medical School Department of Parasitology, Aydın, Turkey; 5 Ege University, Medical School Department of Medical Biology, Bornova, Izmir, Turkey; Institut Pasteur de Tunis, Tunisia

## Abstract

Human visceral leishmaniasis (VL) caused by *L. infantum* and cutaneous leishmaniasis (CL) caused by *L. tropica* and *L. infantum* have been reported in Turkey. *L. infantum* is also responsible for canine leishmaniasis (CanL) and it is widely common in the country. The main aim of the present study was to design a real-time PCR method based on the internal transcribed spacer 1 (ITS1) region in the diagnosis of all clinical forms of leishmaniasis in Mediterranean, and to identify the species directly from clinical samples. Totally, 315 clinical specimens, human/canine visceral (blood, bone marrow, lymph node) and cutaneous (lesion aspiration) samples, and 51 Turkish *Leishmania* isolates typed by isoenzymatic method were included in the study. For optimization, DNA samples of the 34 strains were amplified by conventional ITS1-PCR and then sequenced for designing the primers and probes, allowing the species identification. Following the validation with the isolates, the test was applied on clinical samples and melting temperatures were used for genotyping. A group of PCR products were further sequenced for confirmation and assigning the inter- and intraspecies heterogeneity. The diagnosis of leishmaniasis is successfully achieved by the new real-time PCR method, and the test identified 80.43% of human and canine VL samples as *L.infantum* and 6.52% as *L.tropica*; 52.46% of CL samples as *L. infantum* and 26.90% as *L. tropica*. In 13.04% of visceral and 20.62% of cutaneous samples, two peaks were observed. Hovewer, the higher peak was found to be concordant with the sequencing results in 96.96%, in terms of species identification. The real-time ITS1 PCR assay clearly identified the leishmanial species in 81.58% of all clinical samples. Genotypic variations of *Leishmania* parasites in Turkey within species and intraspecies were observed, and *L. tropica* is also found as causative agent of human and canine VL in Turkey.

## Author Summary

Leishmaniasis caused by *Leishmania* parasites are seen as cutaneous (CL) and visceral (VL) clinical forms in Turkey. *Leishmania* (*L.*) *tropica* and *L. infantum* were determined as CL agents, while *L. infantum* was incriminated for VL in the country. Canine leishmaniasis (CanL) is widely common throughout the country and *L. infantum* is the responsible agent of the disease. Related to *Leishmania* species diversity and different clinical forms in human and dogs in this geographical area, the identification of the parasite species prefers to be done during the time of diagnosis. Internal transcribed spacer region was chosen as the target area for developing a real-time PCR assay to use as a fast and standardized diagnostic method and species identification simultaneously. Clinical samples from parasitologically/serologically proven cases and isolates were included the study, and high positivity rate in species identification was obtained. The method can also determine the intragenomic heterogeneity in *Leishmania tropica* and *L. infantum*. The assay presents a sufficient sensitivity for fast and correct detection of leishmaniasis directly from clinical materials. *L. tropica* and *L. infantum* were found as causative agents of human CL, VL and CanL in Turkey. Knowledge about differences in the parasites is useful for future studies in Turkey.

## Introduction

Leishmaniases is a group of diseases caused by more than 20 species of the protozoan genus *Leishmania* in 98 countries and regions with 350 million people living at risk. The main forms of human disease are visceral (VL), cutaneous (CL) and mucocutaneous (MCL) leishmaniasis [1], [2].

Turkey is of special epidemiological interest because it lies at the crossroad between Asia and Europe and it comprises seven geographical regions with environmental and ecological differences. Human leishmaniasis, both visceral (>40 cases yearly) and cutaneous (>2000 cases yearly) have been reported for centuries in Turkey. Two species of *Leishmania* are prevalent in Turkey, causing anthroponotic CL (*Leishmania tropica*), and zoonotic VL (*Leishmania infantum*) [3], [4]. There is also a single report about the occurrence of *L. major* variants in specific regions of the country [5].

Human VL and CanL are endemic throughout Mediterranean, Ege, Marmara and Black Sea Regions of western Turkey while sporadic in other regions with higher infection rates in dog populations than human cases. There were two zymodemes of *L. infantum* (MON1 and MON-98) in dog isolates while all human VL isolates were identified as *L. infantum* MON-1 by isoenzyme analyses [6]–[8].

Anthroponotic CL caused by *L. tropica* is highly endemic in the Southeastern Anatolia, East Mediterranean and Ege Regions of Turkey [3], [6]–[9]. In the South Anatolia, *L. infantum* in addition to *L. tropica* has also been reported as a causative agent for human CL [6], [10], [11]. *Leishmania major* is known to be endemic in the countries bordering Turkey to the south: Syria, Iraq and Iran [6]. Parasites isolated from CL patients in Sanliurfa province located in Southeastern Region were all identified as *L. tropica* MON304 while in Aydin province located in western part, Ege region, were identified as *L. tropica* MON303 (87%) and *L. tropica* MON304 (13%) [unpublished data].

A universal PCR method targeting the internal transcribed spacer 1 (ITS1) region between the SSU and 5.8S rRNA genes were described for the direct diagnosis of different clinical manifestations of leishmaniasis and parasite identification. This method is applicable where several more than one parasite species are aetiologically relevant. It is highly specific and sensitive detecting approximately 0.2 parasites per sample [12]. Most of the medically important *Leishmania* species are then readily distinguished by DNA sequencing or restriction enzyme analysis of the PCR product. ITS1 PCR restriction fragment length polymorphism (RFLP) are used for direct species identification in patient tissues, blood or other samples without prior parasite culturing, microscopic analysis or other technique [6], [12]–[14].

In endemic areas, the presence of multiple *Leishmania* species with overlapping clinical features and geographical distribution requires the development of sensitive laboratory tests with *Leishmania* species identification in order to evaluate the prognosis of human and canine leishmaniasis and to choose appropriate therapies. Species identification will also contribute to better understanding the epidemiology of leishmaniases in Turkey [15], [16]. Species identification of the agents of leishmaniasis in Turkey is crucial, since the country comprises seven geographical regions with environmental and ecological differences. ITS1 PCR RFLP was successfully performed for clinical samples collected from human leishmaniasis and CanL cases from Turkey [6].

The main aim of the present study was to design a real time PCR method based on internal transcribed spacer 1 (ITS1) region in the diagnosis of all clinical forms of leishmaniasis and identifying of parasite directly from clinical samples (human and canine) or *Leishmania* isolates. We further sequenced the PCR products for confirmation and assigning the inter- and intraspecies heterogeneity.

## Materials and Methods

### Clinical samples and *Leishmania* isolates

The study was carried out in two steps; (a) designing primer/probes which are specific in genus and species level and optimization of a novel ITS1 real time PCR method using Turkish *Leishmania* strains previously identified by multilocus enzyme electrophoresis (MLEE) technique in Montpellier Reference Center and four international reference strains; (b) validation of the method using isolates and different types of clinical samples obtained from only confirmed human and dog leishmaniasis cases. Four international reference controls, *L. infantum/chagasi* (MHOM/XX/1999/LRC-L774), *L. donovani* (MHOM/IN/1980/DD8), *L. tropica* (MHOM/IL/1990/LRC-L590 and MHOM/IL/1996/LRC-L691) and *L. major* (MHOM/IL/2000/LRC-L779) are included.

**Figure 1:**
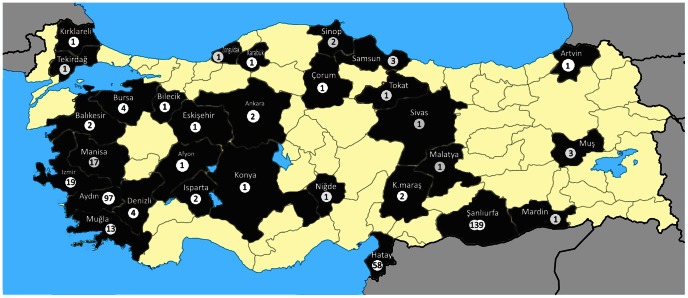
The Turkey map showing the location and the number of samples in province level.

A total of 51 Turkish *Leishmania* strains were isolated from 5 VL, 38 CL, 8 CanL cases between 2000 and 2011, and maintained by subcultures in NNN medium. They were identified by the isoenzymatic method previously. *Leishmania* promastigotes of all isolates were mass cultivated in RPMI+20%FCS medium and centrifuged in 5^th^ day to obtain pellet in order to use DNA extraction The concentration of isolates was adjusted to 2.5–3×10^6^ promastigotes/mL. A total of 315 clinical samples obtained from cases with human CL (n = 223), human VL (n = 40) and CanL (n = 52) originated from 31 different provinces (mostly from Izmir, Aydin, Hatay and Şanlıurfa provinces) of Turkey were included in the study. The samples were collected between April 2007 and May 2010 and they were all confirmed cases who are found positive/seropositive by different parasitological/serological methods in our laboratory (Table 1, Figure 1).

**Table 1:** The type and number of the samples included in the study.

Tissue/Isolates	Type of sample	Clinical feature			Total
		VL	CL	CanL	
**Blood**	Blood with EDTA	8	-	43	51
**Bone marrow**	Smear	32	-	-	32
**Skin**	Aspiration fluid, smear, biopsy	-	223	-	223
**Lymph node**	Aspiration fluid	-	-	9	9
**Isolates**	Promastigotes culture	5	38	8	51
	**Total**	**45**	**261**	**60**	**366**

VL: visceral leishmaniasis; CL: cutaneous leishmaniasis; CanL: canine leishmaniasis.

Blood and bone marrow samples were taken from hospitalized VL patients, and sent to our laboratory in tubes containing EDTA as anticoagulant or on slides. Two hundred microliter sample was used for DNA extraction. Tissue aspirates from CL patients were collected into syringes containing 0.5 mL of saline, a slide was prepared for DNA extraction and part of which was inoculated into NNN medium for isolating the parasite. All the prepared slides were washed with PBS and then this solution was transferred into a 1.5 ml eppendorf tube. The procedures were performed based on the steps mentioned on the DNA extraction kit (Roche Applied Science) for all samples. Quality and quantity of extracted DNA was analysed by spectrophotometry.

### Primer/probe design and optimization

Conventional ITS1-PCR was applied to 30 Turkish *Leishmania* strains (obtained from 18 CL, 5 VL, 7 CanL cases) and four international reference strains using the primer set (forward – LITSR; reverse - L5.8S) and conditions published by El Tai et al [17], [18]. PCR products were sequenced commercially by RefGen (http://www.refgen.com) and compared by multialignment analysis within each other and with other ITS1 sequences published in BLAST using MultAlin program (CLC Main Workbench (v.5.6) genetic program). The forward primer (LITSR) used in the initial experiment was kept but reverse primer (ITS1R-TR1: 5′- GAAGCCAAGTCATCCATCGC -3′) and the probes (Probe1 : CCGTTTATACAAAAAATATACGGCGTTTCGGTTT—FL; Probe 2: LC640-GCGGGGTGGGTGCGTGTGTG—PH) were newly designed according to the variable region for detecting *L. donovani complex, L. tropica and L. major*, using LightCycler Probe Design Software 2.0 program [19].

### The detection of *Leishmania* DNA in clinical samples and differentiation of species

The real time PCR method targeting ITS1 region between the SSU and 5.8S rRNA genes specific for *Leishmania* was first applied to 51 Turkish and international isolates to determine the melting temperatures (Tm) for each species. Then, the method was performed using clinical samples and it was repeated twice for each batch of samples. One positive and two negative controls were included for each PCR reaction.

ITS1 real time PCR method was applied using samples containing 20–50 ng of genomic DNA, 400 nM of each primers , 200 nM of each probes, 2 mM of MgCl_2_, 1 µl LightCycler FastStart DNA Master Hybridisation probe (Roche Applied Science), and 1,5 µl PCR grade water (Roche Applied Science) to a reaction total volume of 10 µl. PCR amplification was performed as follows: one cycle of 10 minutes at 95°C, followed by 45 cycles consisting of denaturation at 95°C for 10 seconds, annealing at 50°C for 10 seconds, extension at 72°C for 20 seconds, and melting at 95°C for 0 second, 50°C for 10 second, 40°C for 10 second, 80°C for 0 second and cooling at 40°C for 30 seconds. Melting curves were analysed using channel 2 and 3.

### Sequencing and phylogenetic analyses

A group of PCR products from 135 samples (121 human; 14 dogs) including 93 clinical specimens and 42 Turkish *Leishmania* isolates were sequenced commercially by RefGen (http://www.refgen.com) for the confirmation of the results. The clinical samples group was consisted of DNA samples from 111 lesion aspiration samples of CL patients, 4 blood and 6 bone marrow samples of VL patients; 12 lymph node aspiration and 2 blood samples of dogs. Sequence data were analysed using ClustalW2 (http://www.ebi.ac.uk/Tools/msa/clustalw2/) program and distance of molecular relationship in the group of samples detected as *L. tropica* and *L. infantum* were calculated. Phylogram was generated using CLC Main Workbench (v.5.6) genetic program.

### Statistical analysis

Univariant and Tukey analyses were performed using SPSS v.15 program in order to compare the melting temperatures in the group of samples detected as *L. tropica* and *L. infantum*. Statistical significance degree was accepted as <0.05.

### Ethical aspects

The study was approved by Local Animal Care and Ethics Committee of the School of Medicine and Ege University Medical School Clinical Research Ethical Committee, Izmir, Turkey.

## Results

### Optimization using clinical *Leishmania* isolates

**Figure 2:**
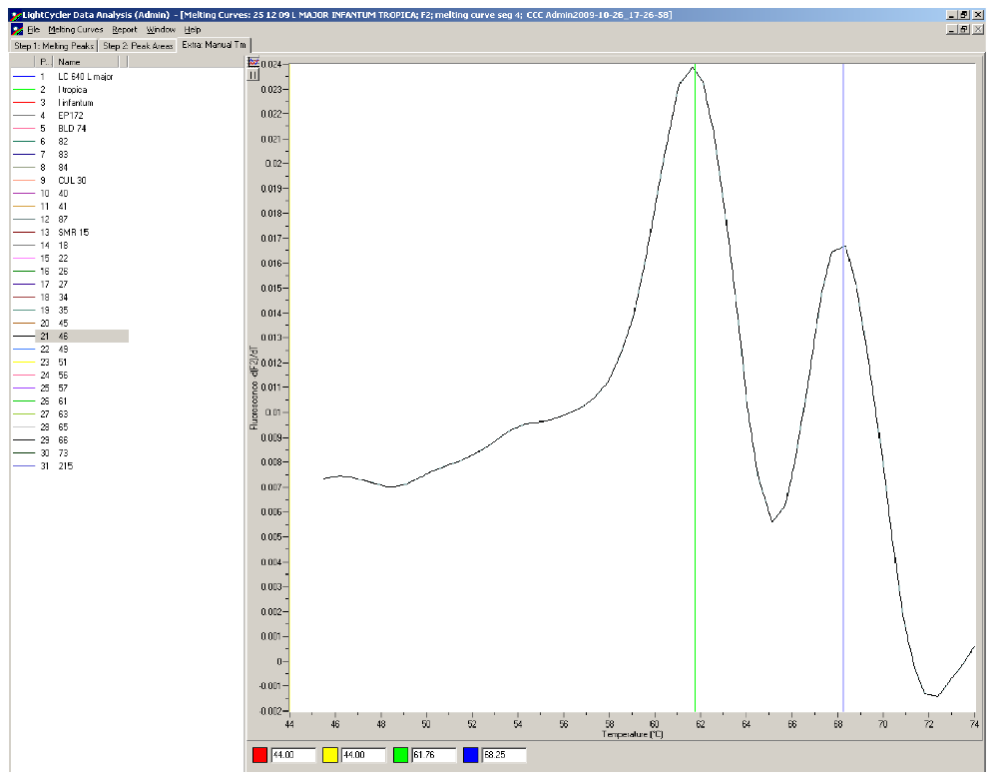
The representative figure of two peaks obtained in some samples.

The melting temperatures were detected as 68°C Tm for *L. donovani* complex; 62°C Tm for *L. tropica* and 53°C Tm for *L. major*. The sequences of variable region which were used for designing probes in Turkish *Leishmania* isolates were shown in figure S1. The identification results of 51 Turkish *Leishmania* isolates were presented with the results of MLEE comparatively in table S1. Result of the isoenzymatic method was used as gold standard. As indicated in the table S1, species identification of 48 isolates well matched with MLEE results, and sensitivity of the method was found to be 94.11%. Out of three isolates which are not fully in aggrement with MLEE, two strains (C010 and C056) are found to be *L. tropica* with PCR while they both were *L. infantum* by MLEE. One strain (C078) gave two peaks with the higher related to *L. tropica* (Figure 2) as concordant with the MLEE analysis (*L. tropica* MON312).

**Figure 3:**
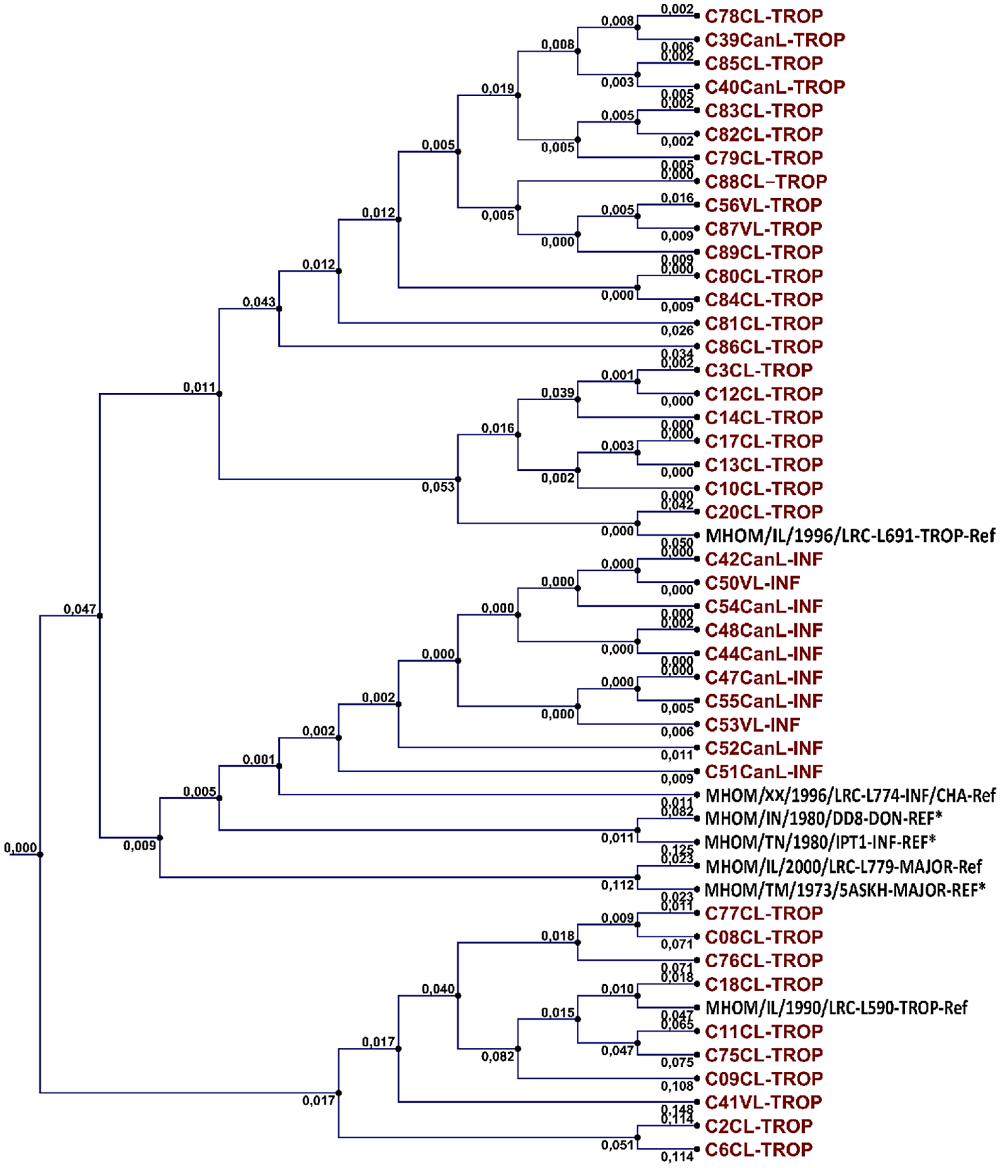
Phylogenetic tree based on the alignment of the amplified section of the ITS1 region of 42 Turkish *Leishmania* isolates and international reference strains (for *L. tropica* MHOM/IL/1990/LRC-L590 and MHOM/IL/1996/LRC-L691; for *L. major* MHOM/IL/2000/LRC-L779; *for L. infantum/chagasi* MHOM/XX/1999/LRC-L774). REF*: ITS1 sequences for *L. infantum* MHOM/TN/1980/IPT1; for *L. donovani* MHOM/IN/1980/DD8; for *L. major* MHOM/TM/1973/5ASKH were taken from Talmi-Frank et al. [47] and included the tree. (VL: visceral isolates; CL: cutaneous isolates; CanL: dog isolates).

Sequencing results of the 42 strains (10 *L. infantum* and 32 *L. tropica*) were used to construct phylogenetic tree. *L. infantum* isolates constituted a single group while strains determined as *L. tropica* showed several different groups (Figure 3).

The partial sequences of *L. tropica* ITS1 region representing MON200, MON303 and MON304 zymodems were submitted to GenBank (Accession numbers KC686338, KC679052 and KC609747, respectively).

### Identification of *Leishmania* in clinical samples

Totally, 315 clinical samples were analysed and all of them were diagnosed as positive by ITS1 real time PCR method in genus level. In species level, *L. infantum* and *L. tropica* were detected while no *L. major* was found among clinical samples. Genotyping identified 80.43% (74/92) of human and canine visceral leishmaniasis samples as *L. infantum* and 6.52% (6/92) as *L. tropica*; 52.46% (117/223) of cutaneous samples as *L. infantum* and 26.90% (60/223) as *L. tropica*.

Fifty-eight (18.41%) out of 315 clinical samples (12 of visceral and 46 of cutaneous samples) gave two peaks and two melting temperatures were observed. The standart deviation of melting temperatures was found between 0.4512 and 0.5026 in one peak samples while it was between 0.3718 and 1.0858 in two peak samples.

The sequencing was done for 102 samples (3 blood and 1 bone marrow samples of VL patients; 2 blood samples of dogs; 56 lesion aspiration samples of CL patients; 40 strains from CL, VL and CanL cases) giving one peak and 33 samples (1 blood samples of VL patient; 1 dog blood sample; 29 lesion aspiration samples of CL patients and 2 strains from CL cases) giving two peaks. The sequencing results were concordant with the real time ITS1 PCR results in 98.03% (100/102) samples. For the two peaks samples, a 96.96% (32/33) concordance were detected between the higher peak in the real time ITS1 PCR and sequencing results indicating that the higher peak can be decisive for the species identification.

**Figure 4:**
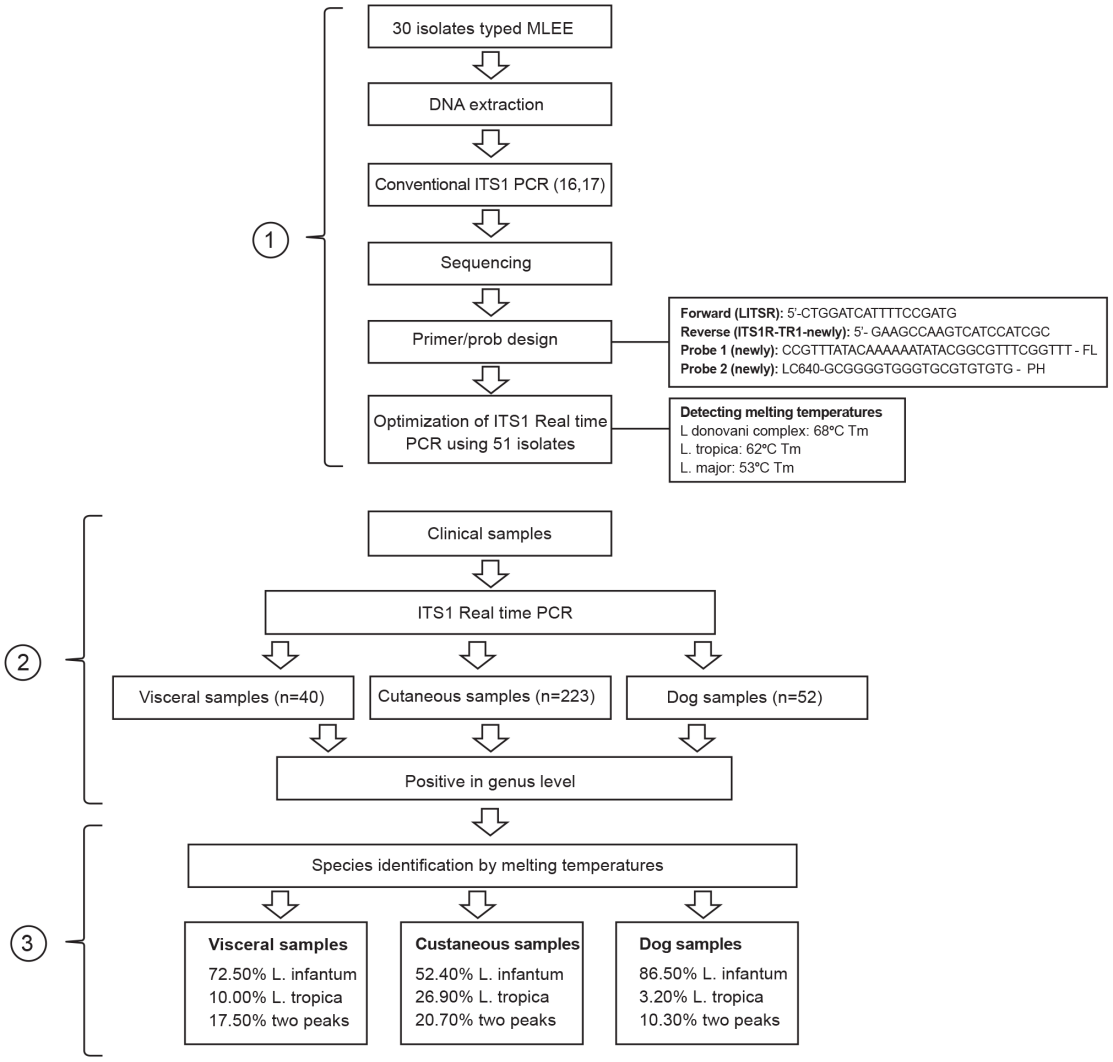
The working chart of the present study. 1: Optimization; 2: Diagnosis; 3: Species identification.

The ITS1 real time PCR results of clinical samples were classified into three groups which were clearly distinguishable as (a) 191 samples diagnosed as *L. infantum* including 29 visceral, 45 dog and 117 cutaneous samples; (b) 66 samples of *L. tropica* including 60 cutaneous and four visceral and two dog samples and (c) 58 samples of two peaks including 46 cutaneous, seven visceral and five dog samples. The working process and the results were summarized in Figure 4. *L. infantum* and *L. tropica* were found to be causative agents of both clinical forms of human leishmaniasis as well as canine leishmaniasis in Turkey.

## Discussion

The various leishmaniases are caused by different species of *Leishmania*, some of which co-exist in the same region like several endemic areas in Turkey; therefore it is crucial to distinguish the species for diagnosis, treatment and epidemiological purposes. Microscopical examination of stained tissue preparations and culture of tissue aspirates for diagnosis, and multilocus enzyme electrophoresis (MLEE) for species identification are accepted as gold standards. However, all the conventional methods applied in the diagnosis of leishmaniasis have medium to low sensitivity and the amplification of DNA by PCR, using different genomic and kDNA targets which is shown to be more sensitive is gradually replacing the traditional methods for the diagnosis of leishmaniasis. Besides, molecular phylogeny studies of the parasite have increasingly suggested new approaches regarding treatment, prognosis of the disease, the distribution of *Leishmania* species in human and animal hosts, as well as in insect vectors for designing appropriate control measures [12], [20]–[22].

Different PCR/PCR-RFLP based methods targeting kinetoplastic DNA, telomeric sequences, gp63, miniexons, β-tubulin, or ribosomal RNA encoding genes (particularly the internal transcribed spacers, ITS) and recently microsatellites, have been proposed for species identification in *Leishmania* parasites using isolates and clinical samples [23], [24]. The ITS1 of the ribosomal DNA repeat unit (rDNA-ITS1) has previously been exploited for Old World *Leishmania* species discrimination using RFLP [12]–[14], reverse hybridization assays [25], and sequencing [26]. There are an estimated 20 to 200 identical copies in the *Leishmania* genome, making it a good target for analyzing low parasite quantities [13], [14], [27]. A new technique applying PCR-SSCP (single-strand conformational polymorphism) was published recently by Chargui et al. The authors notified that SSCP provides more resolution especially when PCR product has weak band on agarose gel and is less expensive than RFLP method [24].

In our study, we present a real-time ITS1-PCR method that can diagnose three Old World *Leishmania* species *L. donovani* complex, *L. tropica* and *L. major*, using newly designed probes to diagnose and simultaneously differentiate between Turkish species in clinical samples. The real time ITS1 PCR assay clearly identified the leishmanial species in 81.58% of all clinical samples. *L. infantum* was identified in 80.43% of human and dog visceral samples while *L. tropica* was detected in 6.52%. In five isolates (C41, C56, C087, C39, C40) from 3 VL patient and 2 dogs, the agent was diagnosed as *L. tropica*. This finding was also supported by isoenzyme typing of the strain C87, resulting as *L. tropica* MON-315 in Montpellier leishmaniasis reference center. This is the first report notifying *L. tropica* as a causative agent of human and canine VL in Turkey although, *L. tropica* has been reported to be isolated from human and canine VL cases in different countries [28]–[34]. Several studies and case reports published from neighbouring country, Iran, showed that *L. tropica* can rarely cause visceral leishmaniasis. Alborzi et al [35] identified only one *L. tropica* among 64 bone marrow/spleen samples while another group also found only one *L. tropica* out of 11 isolates obtained from dogs in Iran [36].

*L. infantum* is reported as a rare causative agent of CL most notably in the Mediterranean Basin countries such as Tunisia, Algeria, Morocco, Spain, Italy, Portugal, Greece and France [2], [37]–[40]. Although *L. tropica* is the main causative agent of CL in Turkey, *L. infantum* has dominancy in the South Anatolia of Turkey, mainly in Hatay and Adana provinces according to identification of *Leishmania* strains by molecular techniques using miniexon gene PCR-RFLP [10], [11], kDNA-PCR [41] and ITS1 PCR-RFLP [6] assays. In our study, it is confirmed that both *Leishmania* species can be found in clinical samples from CL patients in this area. *L. infantum* is also isolated from proven vector sand fly species, *P. tobbi* by Svobodova et al. in the region [11].

So far only little is known about the population structures of the two *Leishmania* species in Turkey and the correlation with geographical origin, biogeographical parameters, clinical outcome, involved animal reservoir (dogs) and the transmitting sand fly species. Several pioneer studies were performed that used PCR-RFLP and/or sequence analysis of the ITS rDNA, minicircle kinetoplast DNA, miniexon, NAGT [5], [6], [10], [42] mainly for species identification. In Turkey, nine zymodemes of *L. tropica* and four of *L. infantum* were described so far [43, unpublished data]. The high degree of heterogeneity in *L. tropica* species has been reported [13], [14], [44] and shown by MLEE [30]. This variation was reported in different level in Old World *Leishmania* species by ITS PCR-RFLP analysis from highest to lowest in order of *L. tropica*>*L. aethiopica*>*L. major*>*L. donovani* [44]. A study using PCR-RFLP/sequencing based on ITS1 region was performed in Iran using clinical samples from CL patients and they showed six different genotype groups of *L. tropica* [45]. In our study, the heterogeneity of *L. tropica* was also observed in phylogenetic analysis. *L. infantum* isolates constituted a single group while strains determined as *L. tropica* showed several different groups.

The main difficulty in the present study was to observe two peaks in some samples. It is probably due to genetic variety in ITS1 region. Genetic polymorphism in ITS region of different strains of same *Leishmania* species and possibility of heterogeneity in individual copies was described by El-Tai et al [17]. In a recent study, after digestion of the amplification product with the HaeIII, the ITS1 PCR assay clearly identified the leishmanial species in CL samples in only 72.3% and *L. tropica* was found to be the most dominant [20]. In our study, the failing of species identification in 18.42% of the samples could be either due to the minuteness of the DNA, possibility of a mix infection, hybridization of different species or intraspecies variations in Turkish *Leishmania* parasites as also commented by Kifaya et al. [20]. We also found that there are 6 copies of our probe region containing variable part in ITS1 sequence (data not shown). Therefore, we evaluated that if all copies are identical in the DNA sample one peak was obtained; if not, several copies have one or two bases difference, two peaks were obtained but always one peak is higher. In the case of detection of two peaks, we decided to take higher one for species identification with the support of sequence analysis and it can be acceptable that the identification in species level was done in all clinical samples. Hereby, we can speculate that our assay could also propose intragenomic heterogeneity of particular isolates and/or *Leishmania* DNA samples obtained from clinical materials. The statistical analyses of melting temperatures in the samples having one and two peaks was also performed and standart deviation was found very low in one peak samples than two peak samples. After we compared these results with sequencing results, we also found highest variability in *L. tropica* group. Gelanew et al. performed PCR-RFLP/direct sequencing assay using *L. donovani* strains and the direct sequencing of both strands of ITS1 DNA showed the presence of multiple peaks in the chromatograms, which could possibly have resulted from: (i) the presence of multiple strains or clones of *Leishmania*; (ii) the presence of a hybrid genotype; (iii) intragenomic variation in the multicopy ITS1; or a combination of these [46]. This should be studied furtherly by multiple gene targets and innovative methods like Multilocus Sequence Typing (MLST) of the genes encoding the proteins used for MLEE and Multilocus Microsatellite Typing (MLMT) as well as experimental animal infection studies using Turkish isolates.

Two aims were achieved through the analysis of real time ITS1 PCR products by sequencing of the reference and local strains as designing new probes and proving the sensitivity of the technique at the genus and species identification level. The results of isolates in the optimization step also showed that real time ITS1 PCR results are highly concordant with MLEE analysis (96.07%). We can also propose that the method can identify inter- and intraspecies variability based on ITS1 region but cannot differentiate *L. donovani* complex species within each other like other molecular assays using ITS1 region [26]. In this point, we would like to emphasize that the studies addressed to *L. donovani* complex species (*L. donovani* and *L. infantum*) identification should be planned.

In conclusion, the proposed method presents a sufficient sensitivity for fast and correct diagnosis of leishmaniasis in all type of clinical samples but due to the samples giving two peaks the ability of the method for species identification is limited and needs further analyses. However, the higher peak was always very well matched with the results of sequence analysis. Genotypic variations based on ITS1 region of *Leishmania* parasites in Turkey within species and intraspecies were determined. The findings in this study were showed that *L. tropica* is one of the causative agents of human and canine visceral leishmaniasis in Turkey.

## Supporting Information

Figure S1 The ITS1 region including the variable region used for designing probes in Turkish Leishmania isolates. [REF*: the sequences were taken from published paper, Talmi-Frank et al. (47); VL: visceral isolates; CL: cutaneous isolates; CanL: dog isolates].
(TIF)

Figure S2 STARD Flowchart of the present study.
(TIF)

Table S1 Identification of Turkish *Leishmania* isolates by ITS1 real time PCR method and isoenzymatic method.
(DOCX)

Table S2 STARD checklist for reporting of the study.
(DOCX)

## References

[1] Desjeux P (2004) Leishmaniasis: Current Situation and New Perspectives. Comp Immunol Microbiol Infect Dis 27: 305–318. 15225981 10.1016/j.cimid.2004.03.004

[2] WHO (2010) Control of the leishmaniasis: report of a meeting of the WHO Expert Committee on the Control of Leishmaniases. World Health Organ Tech Rep Ser 949, Geneva.

[3] Ok UZ, Balcioglu IC, Taylan Ozkan A, Ozensoy S, Ozbel Y (2002) Leishmaniosis in Turkey. Acta Trop 84: 43–48. 12387909 10.1016/s0001-706x(02)00134-1

[4] Alvar J, Vélez ID, Bern C, Herrero M, Desjeux P, et al (2012) Leishmaniasis worldwide and global estimates of its incidence. PLoS One 7: e35671 doi: 10.1371/journal.pone.0035671. 22693548 10.1371/journal.pone.0035671 PMC3365071

[5] Akman L, Aksu HS, Wang RQ, Ozensoy S, Ozbel Y, et al (2000) Multi-site DNA polymorphism analyses of Leishmania isolates define their genotypes predicting clinical epidemiology of leishmaniasis in a specific region. J Eukaryot Microbiol 47: 545–554. 11128706 10.1111/j.1550-7408.2000.tb00088.x

[6] Toz SO, Nasereddin A, Ozbel Y, Ertabaklar H, Culha G, et al (2009) Leishmaniasis in Turkey: Molecular Characterization of Leishmania from Human and Canine Clinical Samples. Trop Med Int Health 14: 1–6. 19737374 10.1111/j.1365-3156.2009.02384.x

[7] Ozensoy S, Ozbel Y, Turgay N, Alkan MZ, Gul K, et al (1998) Serodiagnosis and Epidemiology of Visceral Leishmaniosis in Turkey. Am J Trop Med Hyg 59: 363–369. 9749626 10.4269/ajtmh.1998.59.363

[8] Ozbel Y, Turgay N, Ozensoy S, Ozbilgin A, Alkan MZ, et al (1995) Epidemiology, Diagnosis and Control of Leishmaniosis in the Mediterranean Region. Ann Trop Med Parasitol 89 ((Suppl1)) 89–93. 10.1080/00034983.1995.11813018 8745931

[9] Ertabaklar H, Ozensoy Toz S, Sakru N, Keles E, Ozbel Y (2001) Epidemiology of Visceral Leishmaniasis in the City of Mugla, Turkey. Turkiye Parazitol Derg 25: 128–131.

[10] Serin MS, Daglioglu K, Bagirova M, Allahverdiyev A, Uzun S, et al (2005) Rapid diagnosis and genotyping of *Leishmania* isolates from cutaneous and visceral leishmaniasis by microcapillary cultivation and polymerase chain reaction–restriction fragment length polymorphism of miniexon region. Diag Microbiol Infect Dis 53: 209–214. 10.1016/j.diagmicrobio.2005.05.007 16249065

[11] Svobodova M, Alten B, Zidkova L, Dvorak V, Hlavackova J, et al (2009) Cutaneous leishmaniasis caused by *Leishmania infantum* transmitted by *Phlebotomus tobbi* . Int J Parasitol 39: 251–256. 18761342 10.1016/j.ijpara.2008.06.016

[12] Schönian G, Nasereddin A, Dinse N, Schweynoch C, Schallig HDFH, et al (2003) PCR Diagnosis and characterization of *Leishmania* in local and imported clinical samples. Diag Microbiol Infect Dis 47: 349–358. 10.1016/s0732-8893(03)00093-2 12967749

[13] Schönian G, Schnur L, el Fari M, Oskam L, Kolesnikov AA, et al (2001) Genetic heterogeneity in the species *Leishmania tropica* revealed by different PCR-based methods. Trans R Soc Trop Med Hyg 95: 217–224. 11355565 10.1016/s0035-9203(01)90173-7

[14] Schönian G, Fari ME, Lewin S, Schweynoch C, Presber W (2001) Molecular Epidemiology and Population Genetics in *Leishmania* . Med Microbiol Immunol 190: 61–63. 11770112 10.1007/s004300100081

[15] Ben Abda I, de Monbrison F, Bousslimi N, Aoun K, Bouratbine A, et al (2011) Advantages and limits of real-time PCR assay and PCR-restriction fragment length polymorphism for the identification of cutaneous *Leishmania* species in Tunisia. Trans R Soc Trop Med Hyg 105: 17–22. 20926109 10.1016/j.trstmh.2010.09.003

[16] Wortmann G, Hochberg L, Houng HH, Sweeney C, Zapor M, et al (2005) Rapid identification of *Leishmania* complexes by a real-time PCR assay. Am J Trop Med Hyg 73: 999–1004. 16354801

[17] El Tai NO, Osman OF, El Fari M, Presber W, Schönian G (2000) Genetic heterogeneity of ribosomal internal transcribed spacer in clinical samples of *Leishmania donovani* spotted on filter paper as revealed by single-strand conformation polymorphisms and sequencing. Trans R Soc Trop Med Hyg 94: 575–579. 11132393 10.1016/s0035-9203(00)90093-2

[18] El Tai NO, El Fari M, Mauricio I, Miles MA, Oskam L, et al (2001) *Leishmania donovani*: Intraspecific Polymorphisms of Sudanese Isolates Revealed by PCR-Based Analyses and DNA Sequencing,. Exp Parasitol 97: 35–44. 11207112 10.1006/expr.2001.4592

[19] van der PUT NMJ, Gabreels F, Stevens EMB, Smeitink JAM, Trijbels FJM, et al (1998) A Second Common Mutation in the Methylenetetrahydrofolate Reductase Gene: An Additional Risk Factor for Neural-Tube Defects? Am J Hum Genet 62: 1044–1051. 9545395 10.1086/301825 PMC1377082

[20] Kifaya A, Abedelmajeed N, Ereqat S, Schnur L, Schonian G (2011) Methods incorporating a polymerase chain reaction and restriction fragment length polymorphism and their use as a ‘gold standard’ in diagnosing Old World cutaneous leishmaniasis. Diag Microbiol Infect Dis 71: 151–155. 10.1016/j.diagmicrobio.2011.06.004 21840670

[21] Pratlong F, Dereure J, Ravel C, Lami P, Balard Y, et al (2009) Geographical distribution and epidemiological features of Old World cutaneous leishmaniasis foci, based on the isoenzyme analysis of 1048 strains. Trop Med Int Health 14: 1071–1085. 19624480 10.1111/j.1365-3156.2009.02336.x

[22] Schönian G, Mauricio I, Cupolillo E (2010) Is it time to revise the nomenclature of *Leishmania*? Trends Parasitol 26: 466–469. 20609626 10.1016/j.pt.2010.06.013

[23] Vega-Lopez F (2003) Diagnosis of Cutaneous Leishmaniasis,. Current Opinion in Infectious Diseases 16: 97–101. 12734442 10.1097/00001432-200304000-00006

[24] Chargui N, Haouas N, Jaouadi K, Gorcii M, Pratlong F, et al (2012) Usefulness of a PCR-based method in the detection and species identification of *Leishmania* from clinical samples. Pathol Biol 60: e75–79 doi:10.1016/j.patbio.2011.11.011. 22326417 10.1016/j.patbio.2011.11.011

[25] Nasereddin A, Bensoussan-Hermano E, Schonian G, Baneth G, Jaffe CL (2008) Molecular diagnosis of Old World cutaneous leishmaniasis and species identification by use of a reverse line blot hybridization assay. J Clin Microbiol 46: 2848–2855. 18614659 10.1128/JCM.00951-08 PMC2546705

[26] Talmi-Frank D, Jaffe CL, Nasereddin A, Warburg A, King R, et al (2010) *Leishmania tropica* in rock hyraxes (*Procavia capensis*) in a focus of human cutaneous leishmaniasis. Am J Trop Med Hyg 82: 814–818. 20439960 10.4269/ajtmh.2010.09-0513 PMC2861385

[27] Odiwuor SOC, Saad AA, De Doncker S, Maes I, Laurent T, et al (2011) Universal PCR assays for the differential detection of all Old World *Leishmania* species. Eur J Clin Microbiol Infect Dis 30: 209–218. 20936316 10.1007/s10096-010-1071-3

[28] Schnur LF, Chance ML, Ebert F, Thomas SC, Peters W (1981) The biochemical and serological taxonomy of visceralizing *Leishmania* . Ann Trop Med Parasitol 75: 131–144. 10.1080/00034983.1978.11719357 736662

[29] Schnur LF (1989) On the clinical manifestations and parasites of old world leishmaniases and Leishmania tropica causing visceral leishmaniasis. In: Hart DT, editor. Leishmaniases. NATO Advanced'Scientific Institute Series. New York: Plenum Press. pp. 939–943.

[30] Le Blancq SM, Peters W (1986) Leishmania in the Old World: 2. Heterogeneity among *L. tropica* zymodemes. Trans R Soc Trop Med Hyg 80: 113–119. 3726972 10.1016/0035-9203(86)90208-7

[31] Dereure J, Rioux J-A, Gallego M, Perieres J, Pratlong F, et al (1991) *Leishmania tropica* in Morocco: infection in dogs. Trans R Soc Trop Med Hyg 85: 595. 1780983 10.1016/0035-9203(91)90356-4

[32] Kreutzer RD, Grogl M, Neva FA, Fryauff DJ, Magill AI (1993) Identification and genetic comparison of leishmanial parasites causing viscerotropic and cutaneous disease in soldiers returning from operation Desert Storm. Am J Trop Med Hyg 49: 357–363. 8372957 10.4269/ajtmh.1993.49.357

[33] Magill AJ, Grögl M, Gasser RA Jr, Sun W, Oster CN (1993) Visceral infection caused by *Leishmania tropica* in veterans of Operation Desert Storm. N Engl J Med 328: 1383–1387. 8292114 10.1056/NEJM199305133281904

[34] Guessous-Idrissi N, Berrag B, Riyad M, Sahibi H, Bichichi M (1997) *Leishmania tropica*: etiologic agent of a case of canine visceral leishmaniasis in northern Morocco. Am J Trop Med Hyg 57: 172–173. 9288811 10.4269/ajtmh.1997.57.172

[35] Alborzi A, Rasouli M, Shamsizadeh A (2006) *Leishmania tropica* isolated patient with visceral leishmaniasis in southern Iran. Am J Trop Med Hyg 74: 306–307. 16474088

[36] Mohebali M, Hajjaran H, Hamzavi Y, Mobedi I, Arshi S, et al (2005) Epidemiological aspects of canine visceral leishmaniosis in the Islamic Republic of Iran. Vet Parasitol 129: 243–251. 15845279 10.1016/j.vetpar.2005.01.010

[37] Gramiccia M, Gradoni L (1989) Successful *in vitro* isolation and cultivation of Italian dermotropic strains of *Leishmania infantum* sensu lato. Trans R Soc Trop Med Hyg 83: 76. 2603211 10.1016/0035-9203(89)90713-x

[38] Rioux JA, Lanotte G (1990) *Leishmania infantum* as a cause of cutaneous leishmaniasis. Trans R Soc Trop Med Hyg 84: 898. 10.1016/0035-9203(90)90120-4 2096532

[39] Ben-Ismail R, Smith DF, Ready P, Ayadi A, Gramiccia M, et al (1992) Sporadic cutaneous leishmaniasis in north Tunisia: Identification of the causative agent as *Leishmania infantum* by the use of a diagnostic deoxyribonucleic acid probe. Trans R Soc Trop Med Hyg 86: 508–510. 1475816 10.1016/0035-9203(92)90087-s

[40] Lemrani M, Nejjar N, Benslimane A (1999) A new focus of cutaneous leishmaniasis due to *Leishmania infantum* in Northern Morocco. Giornale Italiano di Medicina Tropicale 4: 3–4.

[41] Culha G, Uzun S, Ozcan K, Memisoglu HR, Chang KP (2006) Comparison of Conventional and Polymerase Chain Reaction Diagnostic Techniques for Leishmaniasis in the Endemic Region of Adana, Turkey. Int J Dermatol 45: 569–572. 16700794 10.1111/j.1365-4632.2006.02695.x

[42] Serin MS, Waki K, Chang KP, Aslan G, Direkel S, et al (2007) Consistence of miniexon polymerase chain reaction-restriction fragment length polymorphism and single-copy gene sequence analyses in discriminating Leishmania genotypes. Diagn Microbiol Infect Dis 57: 295–299. 17141456 10.1016/j.diagmicrobio.2006.09.001

[43] Gouzelou E, Haralambous C, Amro A, Mentis A, Pratlong F, et al (2012) Multilocus microsatellite typing (MLMT) of strains from Turkey and Cyprus reveals a novel monophyletic L. donovani sensu lato group. PLoS Negl Trop Dis 6: e1507. 22348162 10.1371/journal.pntd.0001507 PMC3279343

[44] Gomez-Marin JE (2005) DNA Fingerprinting in Protozoan Infectious Diseases, In: Read MM, editor. Trends in DNA Fingerprinting Research. Hauppauge: NOVA Science Publishers Inc. pp. 62–65.

[45] Doudi M, Ghasemi F, Setorki M (2012) Genetic polymorphism analysis of *Leishmania tropica* isolated from three endemic regions (Bam, Kermanshah and Mashhad) in Iran by PCR-RFLP technique and based on ITS1 sequences. African J Microbiol Res 6: 2970–2975.

[46] Gelanew T, Kuhls K, Hurissa Z, Weldegebreal T, Hailu W, et al (2010) Inference of population structure of *Leishmania donovani* strains isolated from different Ethiopian visceral leishmaniasis endemic areas. PLoS Negl Trop Dis 4: e889. 21103373 10.1371/journal.pntd.0000889 PMC2982834

[47] Talmi-Frank D, Nasereddin A, Schnur LF, Schönian G, Töz SO, et al (2010) Detection and Identification of Old World Leishmania by High Resolution Melt Analysis. PLoS Negl Trop Dis 4: e581. 20069036 10.1371/journal.pntd.0000581 PMC2797090

