# Supplementary figures and images for: A Real-Time ITS1-PCR Based Method in the Diagnosis and Species Identification of Leishmania Parasite from Human and Dog Clinical Samples in Turkey
Source: PLoS Negl Trop Dis. 2013 May 9;7(5):e2205. doi: 10.1371/journal.pntd.0002205 (PMC3649959; doi:10.1371/journal.pntd.0002205)

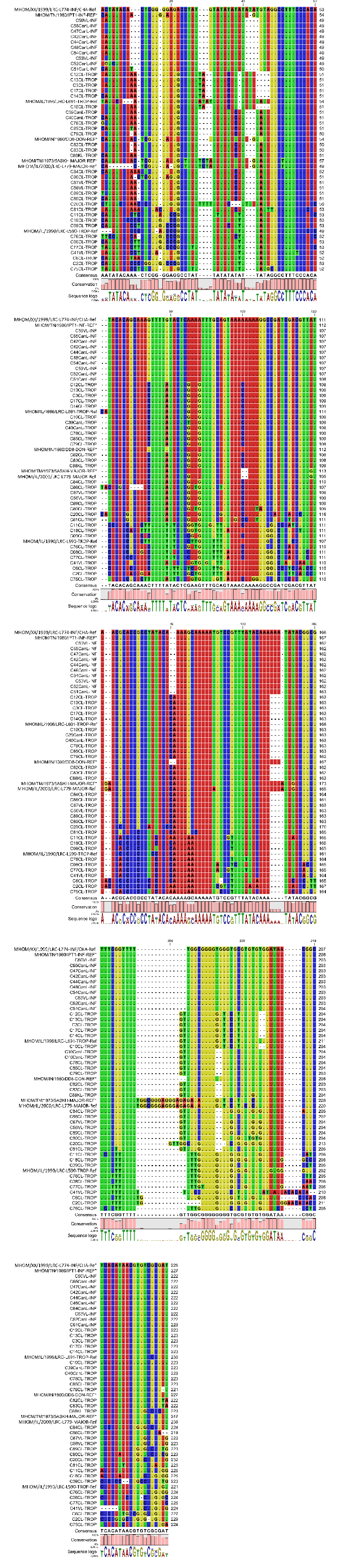

Supplement: Figure S1 — The ITS1 region including the variable region used for designing probes in Turkish Leishmania isolates. [REF*: the sequences were taken from published paper, Talmi-Frank et al. (47); VL: visceral isolates; CL: cutaneous isolates; CanL: dog isolates]. (TIF) [file pntd.0002205.s001.tif]

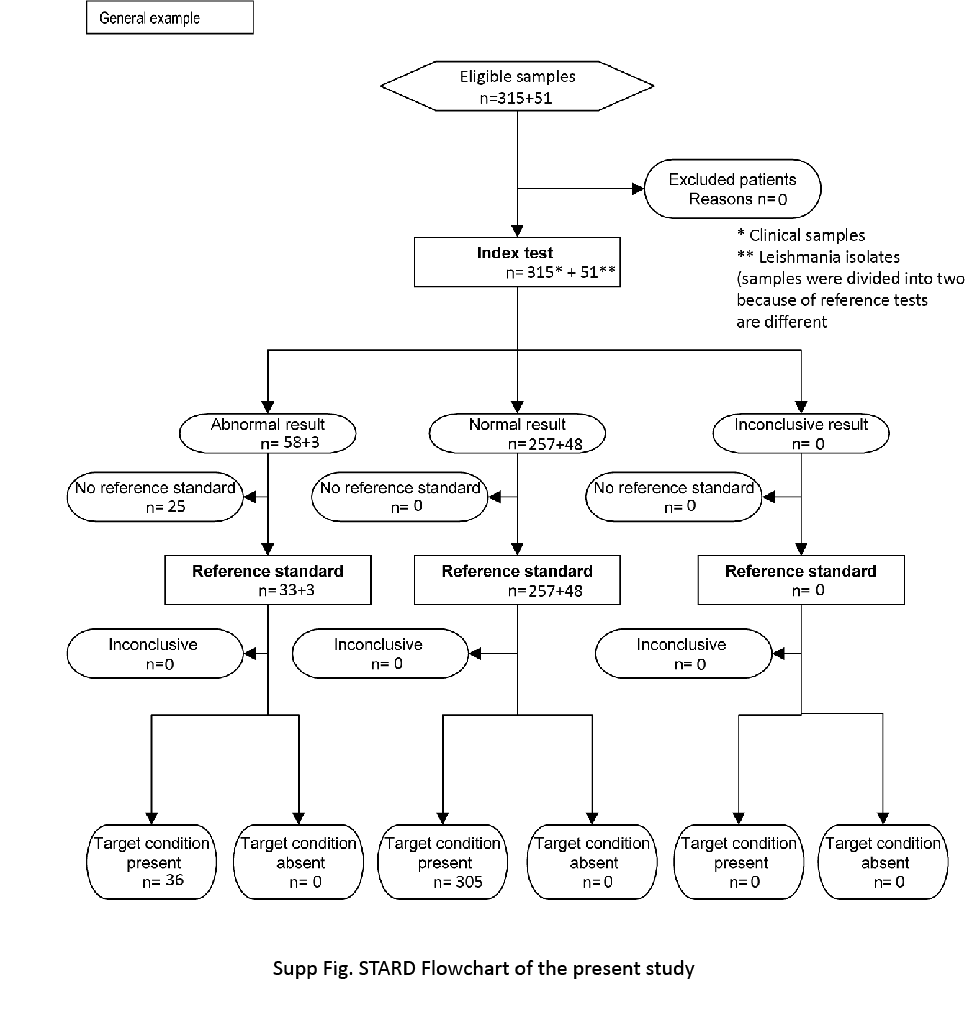

Supplement: Figure S2 — STARD Flowchart of the present study. (TIF) [file pntd.0002205.s002.tif]
