# Supplementary material for: A Real-Time ITS1-PCR Based Method in the Diagnosis and Species Identification of Leishmania Parasite from Human and Dog Clinical Samples in Turkey
Source: PLoS Negl Trop Dis. 2013 May 9;7(5):e2205. doi: 10.1371/journal.pntd.0002205 (PMC3649959; doi:10.1371/journal.pntd.0002205)
Supplement: Table S1 — Identification of Turkish Leishmania isolates by ITS1 real time PCR method and isoenzymatic method. (DOCX) [file pntd.0002205.s003.docx]

**Supp Table 1.** Identification of Turkish *Leishmania* isolates by ITS1 real time PCR method and isoenzymatic method.

| **No** | **Isolate code** | **Clinical feature** | **ITS1 Real time PCR typing** | **Isoenzymatic typing** |
| --- | --- | --- | --- | --- |
| 1 | C003 | CL | *L. tropica* | *L. tropica* MON 200 |
| 2 | C004 | CL | *L. tropica* | *L. tropica* MON 303 |
| 3 | C005 | CL | *L. tropica* | *L. tropica* MON 304 |
| 4 | C006 | CL | *L. tropica* | *L. tropica* MON 304 |
| 5 | C007 | CL | *L. tropica* | *L. tropica* MON 200 |
| 6 | C008 | CL | *L. tropica* | *L. tropica* MON 304 |
| 7 | C009 | CL | *L. tropica* | *L. tropica* MON 304 |
| 8 | C011 | CL | *L. tropica* | *L. tropica* MON 304 |
| 9 | C012 | CL | *L. tropica* | *L. tropica* MON 303 |
| 10 | C015 | CL | *L. tropica* | *L. tropica* MON 304 |
| 11 | C016 | CL | *L. tropica* | *L. tropica* MON 304 |
| 12 | C017 | CL | *L. tropica* | *L. tropica* MON 303 |
| 13 | C018 | CL | *L. tropica* | *L. tropica* MON 304 |
| 14 | C019 | CL | *L. tropica* | *L. tropica* MON 303 |
| 15 | C020 | CL | *L. tropica* | *L. tropica* MON 55 |
| 16 | C021 | CL | *L. tropica* | *L. tropica* MON 304 |
| 17 | C022 | CL | *L. tropica* | *L. tropica* MON 304 |
| 18 | C023 | CL | *L. tropica* | *L. tropica* MON 304 |
| 19 | C024 | CL | *L. tropica* | *L. tropica* MON 304 |
| 20 | C026 | CL | *L. tropica* | *L. tropica* MON 304 |
| 21 | C027 | CL | *L. tropica* | *L. tropica* MON 304 |
| 22 | C028 | CL | *L. tropica* | *L. tropica* MON 303 |
| 23 | C029 | CL | *L. tropica* | *L. tropica* MON 303 |
| 24 | C030 | CL | *L. tropica* | *L. tropica* MON 304 |
| 25 | C031 | CL | *L. tropica* | *L. tropica* MON 304 |
| 26 | C032 | CL | *L. tropica* | *L. tropica* MON 303 |
| 27 | C033 | CL | *L. tropica* | *L. tropica* MON 304 |
| 28 | C034 | CL | *L. tropica* | *L. tropica* MON 304 |
| 29 | C035 | CL | *L. tropica* | *L. tropica* MON 303 |
| 30 | C036 | CL | *L. tropica* | *L. tropica* MON 304 |
| 31 | C037 | CL | *L. tropica* | *L. tropica* MON 303 |
| 32 | C038 | CL | *L. tropica* | *L. tropica* MON 304 |
| 33 | C058 | CL | *L. tropica* | *L. tropica* MON 303 |
| 34 | C065 | CL | *L. tropica* | *L. tropica* MON 304 |
| 35* | C078 | CL | *L. tropica + L. infantum* | *L. tropica* MON 312 |
| 36 | C081 | CL | *L. tropica* | *L. tropica* MON 315 |
| 37 | C088 | CL | *L. tropica* | *L. tropica* MON 315 |
| 38 | C089 | CL | *L. tropica* | *L. tropica* MON 315 |
| 39* | C010 | CanL | *L. tropica* | *L. infantum* MON 98 |
| 40 | C042 | CanL | *L. infantum* | *L. infantum* MON 98 |
| 41 | C044 | CanL | *L. infantum* | *L. infantum* MON 98 |
| 42 | C045 | CanL | *L. infantum* | *L. infantum* MON 98 |
| 43 | C046 | CanL | *L. infantum* | *L. infantum* MON 98 |
| 44 | C047 | CanL | *L. infantum* | *L. infantum* MON 98 |
| 45 | C054 | CanL | *L. infantum* | *L. infantum* MON 1 |
| 46 | C055 | CanL | *L. infantum* | *L. infantum* MON 1 |
| 47 | C087 | VL | *L. tropica* | *L. tropica* MON 315 |
| 48 | C050 | VL | *L. infantum* | *L. infantum* MON 1 |
| 49* | C056 | VL | *L. tropica* | *L. infantum* MON 55 |
| 50 | C090 | VL | *L. infantum* | *L. infantum* MON 1 |
| 51 | C091 | VL | *L. infantum* | *L. infantum*MON 1 |

*unmatched isolates
